# Disinfectant and Antimicrobial Susceptibility Studies of *Staphylococcus aureus* Strains and ST398-MRSA and ST5-MRSA Strains from Swine Mandibular Lymph Node Tissue, Commercial Pork Sausage Meat and Swine Feces

**DOI:** 10.3390/microorganisms9112401

**Published:** 2021-11-22

**Authors:** Ross C. Beier, Kathleen Andrews, Michael E. Hume, Muhammad Umar Sohail, Roger B. Harvey, Toni L. Poole, Tawni L. Crippen, Robin C. Anderson

**Affiliations:** 1 Food and Feed Safety Research Unit, Agricultural Research Service, Southern Plains Agricultural Research Center, U.S. Department of Agriculture, 2881 F&B Road, College Station, TX 77845, USA; kate.andrews@usda.gov (K.A.); michael.hume@usda.gov (M.E.H.); roger.harvey@usda.gov (R.B.H.); toni.poole@usda.gov (T.L.P.); tc.crippen@usda.gov (T.L.C.); robin.anderson@usda.gov (R.C.A.); 2 Proteomics Core, Weill Cornell Medicine, Qatar Foundation—Education City, Doha P.O. Box 24144, Qatar; mus4008@qatar-med.cornell.edu

**Keywords:** antimicrobial, disinfectant, pork sausage meat, *Staphylococcus aureus*, swine, ST398, ST5, swine lymph node tissue

## Abstract

*Staphylococcus aureus* (*S. aureus*) causes gastrointestinal illness worldwide. Disinfectants are used throughout the food chain for pathogenic bacteria control. We investigated *S. aureus* bioavailability in swine Mandibular lymph node tissue (MLT) and pork sausage meat (PSM), established susceptibility values for *S. aureus* to disinfectants, and determined the multilocus sequence type of MRSA strains. Antimicrobial and disinfectant susceptibility profiles were determined for 164 *S. aureus* strains isolated from swine feces (*n* = 63), MLT (*n* = 49) and PSM (*n* = 52). No antimicrobial resistance (AMR) was detected to daptomycin, nitrofurantoin, linezolid, and tigecycline, while high AMR prevalence was determined to erythromycin (50.6%), tylosin tartrate (42.7%), penicillin (72%), and tetracycline (68.9%). Methicillin-resistant *S. aureus* (MRSA) strains, ST398 (*n* = 6) and ST5 (*n* = 1), were found in the MLT and PSM, 4 MRSA in MLT and 3 MRSA strains in the PSM. About 17.5% of feces strains and 41.6% of MLT and PSM strains were resistant to chlorhexidine. All strains were susceptible to triclosan and benzalkonium chloride, with no cross-resistance between antimicrobials and disinfectants. Six MRSA strains had elevated susceptibilities to 18 disinfectants. The use of formaldehyde and tris(hydroxylmethyl)nitromethane in DC&R was not effective, which can add chemicals to the environment. Didecyldimethylammonium chloride and benzyldimethylhexadecylammonium chloride were equally effective disinfectants. ST398 and ST5 MRSA strains had elevated susceptibilities to 75% of the disinfectants tested. This study establishes susceptibility values for *S. aureus* strains from swine feces, mandibular lymph node tissue, and commercial pork sausage against 24 disinfectants. Since it was demonstrated that *S. aureus* and MRSA strains can be found deep within swine lymph node tissue, it may be beneficial for the consumer if raw swine lymph node tissue is not used in uncooked food products and pork sausage.

## 1. Introduction

*Staphylococcus aureus* (*S. aureus*) is a Gram-positive bacterial pathogen important worldwide because of its human health effects [1,2,3,4,5]. Toxic shock syndrome is often caused by toxins produced by *S. aureus* bacteria [6,7], and *S. aureus* is a well-known cause of hospital and community acquired diseases involving the bloodstream, endocarditis, lungs, meningitis, sepsis, skin, and soft tissue infections [8,9,10,11,12,13,14]. The Centers for Disease Control (CDC) has selected *S. aureus* as one of the top five pathogens causing foodborne illness in the United States [15]. Methicillin-resistant *S. aureus* (MRSA) was discovered soon after the first clinical application of the antibiotic methicillin [16]. Methicillin resistance in *S. aureus* results from the acquisition of the *mecA* gene, which codes for a penicillin binding protein (PBP2′ or PBP2a) [4,17]. Therefore, MRSA is resistant to all penicillins [13] and commonly exhibits resistance to most β-lactams [18], such as amoxicillin and oxacillin, resulting in limited treatment options for MRSA infections [18,19]. An emerging *S. aureus* strain referred to as borderline oxacillin-resistant *S. aureus* (BORSA) does not have PBP2a and cannot be either classified as methicillin-resistant or methicillin-susceptible [20]. BORSA resistance results from hyperproduction of beta-lactamases or point mutations. Therefore, they may be misidentified causing a therapeutic threat. MRSA infections are a major problem, especially MRSA-associated bacteremia, which is a cause of endocarditis and sepsis resulting in morbidity and mortality [21].

*S. aureus* can cause foodborne illnesses if it enters the food-chain through contamination by food handlers or preparers [22,23], since people and other animals generally carry *S. aureus* in their nose and on their skin. *S. aureus* contamination may not affect the physical appearance of food, but toxins produced by *S. aureus* can cause food poisoning, leading to gastrointestinal illnesses [24]. Raw milk and raw milk cheese products have resulted in significant *S. aureus* food poisoning outbreaks worldwide [25,26,27,28,29,30]. Additionally, *S. aureus* is routinely found in raw or cooked meat, seafood, and vegetables [26,27,29,31,32]. Ground pork meat [26,27] and sausage [31,33] are commonly associated with gastroenteritis caused by MRSA [34], and staphylococcal food poisoning is an important foodborne disease worldwide [35], which is predominantly caused by *S. aureus* [36]. Beef [32] and pork sausage [32,37] are excellent media to promote the growth of *S. aureus*.

Pigs are routinely colonized by *S. aureus* strains [38] and can be considered a reservoir for *S. aureus* [3,4,39,40,41]. Pig- and other livestock-associated MRSA (LA-MRSA) represents one of the largest MRSA reservoirs outside of the hospital setting [41]. LA-MRSA can be both a donor and recipient of antimicrobial resistance (AMR) genes [42]. The LA-MRSA clone most frequently detected is LA-MRSA clonal cluster 398 (CC398), which contains a wide array of resistance genes observed in numerous *S. aureus* strains [42]. The practice of exposing microorganisms to low levels of antimicrobials is common for growth promotion of food animals and can produce antimicrobial-resistant *S. aureus* [43,44], MRSA [25,45,46,47,48], and multidrug-resistant MRSA (MDRSA) [49,50]. CC398 strains have emerged in areas with a high-density of swine farms [48,51,52,53,54] in the United States [48,49,55] and Europe [4,46,56,57,58]. MRSA CC398 does not have host specificity and readily colonizes and causes infections in humans and other animals [3,59]. As a result, swine carriage of *S. aureus* can lead to occupational exposure to *S. aureus* [49,60,61,62,63,64] its multidrug-resistant MRSA (MDRSA) strains [54,65], and the potential for illness and hospitalization.

Prior to 1995 MRSA infections were predominantly associated with hospitalized patients (HA-MRSA) [54]. However, since the mid-1990s, MRSA has also been observed in community-associated infections (CA-MRSA) [13,66]. During the early 2000s a third type of MRSA from the community began emerging in humans [55] that belonged to CC398 [67]. CC398 is a group of at least five sequence types, each type characterized as sharing identical housekeeping genes [4]. This clonal cluster group is referred to as multilocus sequence type (MLST) 398 (ST398), since ST398 had the largest number of single-locus variants [68]. Thus, the threat imposed by CC398 emanates from the community, health care facilities, and is widely associated with food animal-producing farms.

Pathogenic bacteria that penetrate the food chain can cause foodborne illness. These bacteria can be derived from multiple locations, including from the farm, slaughterhouse, food processing centers, food handlers, and from handling food products within the home. These bacteria can be controlled through biocide (antiseptics and disinfectants) application programs [69,70]. A general definition of disinfectants was presented by White and McDermott [71], stating that disinfectants are chemicals that can kill or inhibit a broad-spectrum of microorganisms. However, if the levels of biocides used are lower than that required to kill the target bacteria, cross-resistance may be exacerbated [69,72,73,74,75,76,77,78,79,80,81,82], and surviving bacteria may develop biofilms resulting in biocide tolerance and increased AMR [70,83,84,85]. The QAC didecyldimethylammonium chloride (DDAC) (referred to as C10AC in our laboratory to indicate the carbon chain length), was found to adsorb physicochemically onto the cell membrane where it could damage and disrupt the *S. aureus* membrane structure and function [86]. Further mode-of-action studies of C10AC and a mixture of *N*-alkylbenzyldimethylammonium chlorides (BACs) against *S. aureus* showed that C10AC formed a double monolayer, and the BACs formed a single monolayer that covered the bacterial cells, resulting in substantial depletion of the potassium pool [87]. A study of the QAC benzalkonium chloride (BKC) against meat-associated *Staphylococcus* spp. demonstrated an open reading frame (ORF) on the plasmid pST827 that was similar to the QAC resistance genes *qacC*, *ebr*, and *smr* [88]. Hydrogen peroxide and sodium hypochlorite [89,90] were more effective against *S. aureus* biofilms than quaternary ammonium chloride (QAC) disinfectants [91]. However, these studies were very limited in the number of *S. aureus* bacteria studied and the number of disinfectants tested [89,90,91]. Previously, our laboratory has also investigated the effects of a wide array of disinfectants on the inhibition of foodborne pathogens, *Escherichia coli* O157:H7 [92], *Pseudomonas aeruginosa* [93], non-O157 Shiga toxin-producing *E. coli* strains (STECs) [94], *Salmonella* spp. [95], *Campylobacter coli* [96], *C. jejuni* [70], and vancomycin-resistant enterococci (VRE) [97]. In all studies C10AC resulted in the best bacterial inhibition by an ammonium chloride except against *C. coli*, in which both C10AC and the BACs appeared to perform equally well, and synergistically in the complex disinfectant P-128 [96].

The current study evaluated the susceptibility of 164 *S. aureus* strains isolated from swine feces, swine mandibular lymph node tissue (MLT), and commercial pork sausage meat (PSM) against 16 antimicrobials, 17 disinfectants, and 7 disinfectant components. Multilocus sequence typing was conducted on the seven MRSA strains found in the MLT and PSM. The disinfectant component susceptibilities in some complex disinfectants were calculated and their individual potencies discussed. The potency of various individual disinfectant component ammonium chlorides with respect to the alkyl carbon chain length of the ammonium chlorides are discussed.

## 2. Materials and Methods

### 2.1. Staphylococcus aureus Strains

The 164 *S. aureus* strains were previously isolated from swine feces (*n* = 63), MLT (*n* = 49), and PSM (*n* = 52) [98]. The isolated bacteria were held at −72 °C until used. Prior to experimentation, each *S. aureus* strain was grown for 24 h at 37 °C on plates containing Trypticase™ Soy Agar with 5% sheep blood (TSA II™) (BD BBL™ Stacker™ Plate, Becton, Dickinson and Company, Sparks, MD, USA).

### 2.2. Susceptibility Testing

Antimicrobial susceptibility testing (AST) and disinfectant susceptibility testing (DST) were performed on the 164 *S. aureus* strains using standard broth microdilution methods according to the Clinical and Laboratory Standards Institute [99,100]. Mueller-Hinton broth had previously been shown to not influence the results of suspension tests with disinfectants and *E. coli* DSM 682 or *S. aureus* ATCC 6538 [101]. The lowest concentration of the antimicrobial that had no visible *S. aureus* growth was determined to be the minimum inhibitory concentration (MIC) [102].

### 2.3. Antimicrobial Susceptibility Testing

AST was used to determine the *S. aureus* MICs against 16 antimicrobials using the National Antimicrobial Resistance Monitoring System (NARMS) Gram-positive plate (CMV3AGPF). The strains were adjusted to the proper concentration using a 0.5 McFarland standard and dilution tubes containing demineralized water (5 mL) obtained from Remel Inc. (Lenexa, KS, USA). The inoculated demineralized water (30 µL) was added to tubes containing 11 mL of cation-adjusted Mueller-Hinton broth (MHB) with TES (Tris, EDTA, and NaCl, pH 8) and dose heads (#E3010) obtained from Remel Inc. were used to inoculate the antimicrobial-containing plates. MICs of the 164 *S. aureus* strains were obtained for 16 antimicrobials (Aminoglycosides: gentamicin (GEN), kanamycin, streptomycin (STR); Amphenicols: chloramphenicol (CHL); Cyclic lipopeptides: daptomycin; Fluoroquinolones: ciprofloxacin (CIP); Glycopeptides: vancomycin; Lincosamides: lincomycin; Macrolides: erythromycin (ERY), tylosin tartrate (TYLT); Nitrofurans: nitrofurantoin; Oxazolidinones: linezolid; Penicillins: penicillin (PEN); Streptogramins: quinupristin/dalfopristin (SYN); and Tetracyclines: tetracycline (TET), tigecycline) by following the manufacturer’s instructions for the Sensititre^®^ susceptibility system (Trek Diagnostic Systems Inc., Independence, OH, USA). *Staphylococcus aureus* ATCC 29213 and *Pseudomonas aeruginosa* ATCC 27853 were used as control strains for AST.

### 2.4. DNA Isolation from MRSA Strains for Molecular Analysis

MRSA strains were determined by traditional cefoxitin and oxacillin susceptibility tests of all isolated *S. aureus* strains followed by confirmation by polymerase chain reaction (PCR) methods [103]. A QIAmp^®^ DNA Mini Kit (51306, Qiagen, Valencia, CA, USA) was used to isolate and purify genomic DNA from colonies of pure culture of *S. aureus* isolates. The colonies were grown on TSA II™ plates for 24 h at 37 °C. A loop-full (10-µL loop) of colonies were collected from the plate. Qiagen Protocol D under Protocols for Bacteria was followed for DNA isolation. For protocol D, 200 µg/mL of lysostaphin (L7386-1MG, Sigma-Aldrich, St. Louis, MO, USA) was used to pre-incubate the Gram-positive *Staphylococcus* cells. A NanoDrop™ One (13400518PR2, Thermo Fisher Scientific, Madison, WI, USA) spectrophotometer was used to obtain DNA concentrations and sample purity. Two-microliters of DNA-containing solution was used for each measurement.

### 2.5. Multilocus Sequence Typing of the Extracted DNA

Genomic DNA was sent to a commercial laboratory for Multilocus Sequence Typing (MLST) (Molecular Research Laboratory, Shallowater, TX, USA). *S. aureus* ATCC^®^ 43300 and *S. aureus* ATCC^®^ 29213 were used as controls for MLST testing. Molecular typing of the *S. aureus* isolates was performed by MLST software version 2.19.0 using Galaxy tools [104]. Alleles of each locus were compared, and sequence types were assigned based on the *S. aureus* MLST database [67].

### 2.6. Disinfectant Susceptibility Testing

In this work, 17 disinfectants and 7 disinfectant components, a total of 24 chemicals were evaluated by DST for inhibition of 164 *S. aureus* isolates [98] by methods similar to those previously described [97]. The sources and recommended uses for 21 of 24 chemicals tested were previously reported [95]. Briefly, a list of the 21 chemical disinfectants and disinfectant components with their abbreviations and with the added exponent “CP” to indicate a commercial product are listed as follows (name, abbreviation): benzalkonium chloride, BKC; Betadine^CP^ (10% povidone-iodine), P-I; cetylpyridinium bromide hydrate, CPB; DC&R^CP^, DC&R^CP^; ethylhexadecyldimethylammonium bromide, CDEAB; Food Service Sanitizer^CP^, FSS; F-25 Sanitizer^CP^, F25; Final Step 512 Sanitizer^CP^, FS512; cetylpyridinium chloride hydrate, CPC; cetyltrimethylammonium bromide, CTAB; Novasan Solution^CP^ (chlorhexidine diacetate), chlorhexidine; Odoban^CP^, Odoban^CP^; P-128^CP^, P-128^CP^; Tek-Trol^CP^, Tek-Trol^CP^; triclosan (ergasan), triclosan; didecyldimethylammonium chloride, C10AC; benzyldimethyldodecylammonium chloride, C12BAC; benzyldimethyltetradecylammonium chloride, C14BAC; benzyldimethylhexadecylammonium chloride, C16BAC; J.T. Baker 37% formaldehyde, formaldehyde; and tris(hydroxylmethyl)nitromethane, THN. The additional three chemicals tested are the following: CaviCide^CP^ is a multi-purpose disinfectant that can be used on the hands as well as to decontaminate non-porous surfaces. CaviCide^CP^ kills bacteria and viruses including but not limited to TB, Norovirus, HIV-1, HBV and HCV [105]. Triclocarban, or 3,4,4′-trichlorocarbanilide (TCC), is used worldwide as an antimicrobial both in personal care products (such as, bar soap, cleansing lotions, and deodorants) [106], and in pharmaceuticals [107]. Both CaviCide^CP^ and TCC were obtained from Sigma-Aldrich (Milwaukee, WI, USA). TCC has been shown to be an endocrine disruptor [108,109]. The chemical, dioctyldimethylammonium chloride (C8AC), is a disinfectant component used in complex disinfectants and was obtained from Lonza Inc. (Fairlawn, NJ, USA). To aid the solubility of some chemicals, dimethyl sulfoxide (DMSO) (MilliporeSigma, St. Louis, MO, USA) was added to purified reverse-osmosis water (^RO^H_2_O) obtained using a water purification system from MilliporeSigma (Bedford, MA, USA).

Many disinfectants have multiple active components, and the percentages of active components in the complex disinfectants used in this study were previously provided [95]. The following disinfectants are mixtures of active components: F25, FS512, FSS, DC&R^CP^, P-128^CP^, and Tek-Trol^CP^. *S. aureus* MICs for the disinfectants containing multiple active components have been determined using the authentic complex disinfectants. Triclosan resistance was determined by using the published susceptible/resistant criterion [110]; *S. aureus* bacteria were considered susceptible at MICs < 0.5 µg/mL, were intermediate with MICs from 0.5 to 2 µg/mL and were considered resistant at MICs > 2 µg/mL triclosan. The breakpoint used for chlorhexidine against *S. aureus* was the same as previously used [111] for staphylococci bacteria; MICs ≥ 1 µg/mL were resistant. The susceptible/resistant criterion used for BKC was previously defined [112]; *S. aureus* at MICs < 30 µg/mL were considered susceptible, MICs from 30 to 50 µg/mL were assigned low-level resistance, and *S. aureus* at MICs > 50 µg/mL were considered resistant to BKC.

Dilutions of disinfectants and disinfectant components were made using ^RO^H_2_O followed by filter sterilization using 0.2 µm × 25 mm syringe filters (#431224, Corning Inc., Corning, NY, USA). Some chemicals were not sufficiently soluble with pure ^RO^H_2_O and required an addition of DMSO to achieve solubility. The chemicals that required added DMSO are listed using the following format: Chemical (% DMSO added, % DMSO in final solution). The chemicals requiring DMSO for solubility were the following: CPB (100%, 5%); CPC (30%, 4.5%); CTAB (100%, 4.5%); C14BAC (20%, 1%); C16BAC (60%, 1.5%); TCC (80%, 2.5%); Tek-Trol^CP^ (93%, 4%); THN (60%, 2.5%); and triclosan (100%, 4%). The final working solutions of chemicals did not contain more than 5% DMSO. The methods used to perform DST of *S. aureus* strains were similar to the DST conducted on *Salmonella* spp. [95] and *Campylobacter coli* [96]. The following concentration gradients were tested against the 164 *S. aureus* strains. BKC, 0.25–256 µg/mL; CaviCide^CP^, 1–1024 µg/mL; chlorhexidine, 0.008–8 µg/mL; CDEAB, 0.016–16 µg/mL; CPB, 0.016–16 µg/mL; CPC, 0.016–16 µg/mL; CTB, 0.016–16 µg/mL; C8AC, 0.062–64 µg/mL; C10AC, 0.062–64 µg/mL; C12BAC, 0.25–256 µg/mL; C14BAC, 0.062–64 µg/mL; C16BAC, 0.062–64 µg/mL; DC&R^CP^, 1–1024 µg/mL; formaldehyde, 2–2048 µg/mL; FSS, 0.062–64 µg/mL; FS512, 0.062–64 µg/mL; F25, 0.062–64 µg/mL; OdoBan^CP^, 0.062–64 µg/mL; P-I, 32–32,768 µg/mL; P-128^CP^, 0.016–16 µg/mL; Tek-Trol^CP^, 0.25–256 µg/mL; triclosan, 0.002–2 µg/mL; THN, 2–2048 µg/mL; and TCC, 0.016–16 µg/mL. *Staphylococcus aureus* ATCC 29213 was used as the control strain for DST.

### 2.7. Calculation of Theoretical MICs for Multiple Component Disinfectants

The following calculations were used to obtain the theoretical MICs (^theo^MICs) for the active components in complex disinfectants. The ^theo^MICs estimate the concentration levels of individual active ingredients in a disinfectant mixture.

#### 2.7.1. Calculation of ^theo^MICs for the Active Components of the Complex Disinfectant DC&R^CP^ against *S. aureus* Strains

The active ingredients in DC&R^CP^ consists of a mixture of three disinfectant components, benzyl ammonium chlorides (BACs) (C12BAC-67%, C14BAC-25% and C16BAC-7% and (C8BAC, C10BAC, and C18BAC)-1%) at 3.08%, formaldehyde (Form) at 2.28%, and THN at 19.2%. The theoretical MICs for each component in DC&R^CP^, ^theo^MIC_BACs_^DC&R^, ^theo^MIC_Form_^DC&R^ and ^theo^MIC_THN_^DC&R^, can be calculated by multiplying each determined DC&R^CP^ MIC by the percentage of each component 3.08, 2.28, and 19.2, respectively, and then dividing the result by the sum of the percentages for all active components in DC&R^CP^, which is 24.56, as previously described [95].

#### 2.7.2. Calculation of ^theo^MICs for the Active Components of the Complex Disinfectant P-128^CP^ against *S. aureus* Strains

The active ingredients in P-128^CP^ consists of a mixture of the BACs (C12BAC-40%, C14BAC-50%, and C16BAC-10%) at 3.38% and C10AC at 5.07%. The ^theo^MICs for the active components of P-128^CP^ can be calculated similarly to the DC&R^CP^ components above. Briefly, the ^theo^MICs^P-128^ of the two active components in P-128^CP^, ^theo^MICs_BACs_^P-128^ and ^theo^MICs_C10AC_^P-128^ can be obtained by multiplying each determined P-128^CP^ MIC by the percentage of each component, 3.38 and 5.07, respectively, and then dividing by the sum of the component percentages in P-128^CP^, which is 8.45.

## 3. Results

### 3.1. Antimicrobial Resistance

Table 1 shows the AMR profiles among the 164 *S. aureus* strains isolated from swine feces, MLT, and PSM. Table 1 provides the MIC_50_, MIC_90_, MIC range of recorded values, the number of resistant strains and breakpoint used for each of the 16 antimicrobials tested against the *S. aureus* strains. No resistant strains were found against four different antimicrobials: daptomycin, nitrofurantoin, linezolid, and tigecycline. A low level of resistant strains was observed for the five antimicrobials: gentamicin (0.6%), streptomycin (2.4%), chloramphenicol (3.8%), vancomycin (0.6%), and quinupristin/dalfopristin (3.7%). There were 21/164 (12.8%) resistant *S. aureus* strains against ciprofloxacin. However, there was an observed high level of resistant strains against the four antimicrobials, erythromycin, tylosin tartrate, penicillin, and tetracycline at percentage levels of 50.6, 42.7, 72, and 68.9%, respectively. When a bacterial strain exhibits a MIC [102] less than the breakpoint value for an antimicrobial it is considered susceptible to the antimicrobial. When the MIC is higher than the breakpoint it is considered resistant to the antimicrobial. The susceptible/resistant determination for *S. aureus* cannot be determined for kanamycin and lincomycin using the CMV3AGPF susceptibility plates since the lowest value for kanamycin on the susceptibility plate is 128 μg/mL but the kanamycin breakpoint is only ≥64 μg/mL. The highest value of lincomycin on the susceptibility plate is 8 μg/mL and is far lower than the lincomycin breakpoint for *S. aureus* (≥32 μg/mL). The individual *S. aureus* AMR profiles for strains from swine feces is presented in Supplementary Table S1, the AMR profiles for the strains from the MLT are in Supplementary Table S2, and the AMR profiles for the strains isolated from the PSM are in Supplementary Table S3.

Resistance profiles for the 164 *S. aureus* strains are provided in Table 2 according to sample type, the number of multidrug-resistant (MDR) stains within each sample type, the number of antimicrobial-resistant strains, and the resistance profiles for each resistant strain. MDR strains are resistant to 3 or more classes of antimicrobials [113]. Out of 63 *S. aureus* strains isolated from swine feces, 32 (50.8%) were shown to be MDR with the overall major resistance profile of ERY-TET-PEN-TYLT. There were 49 *S. aureus* strains isolated from the MLT, and 16 strains (32.7%) were determined to be MDR with 4 (25.0%) of these strains also determined to be MRSA [98]. There was no major resistance pattern in this group, except that MDR strains had seven unique resistance profiles. The two most prevalent resistance profiles found in the MLT MRSA strains were TET-PEN and TET-CIP-PEN. Out of 52 *S. aureus* strains, isolated from the PSM, only 15 strains (28.8%) were MDR, including 3 (20.0%) MRSAs [98]. Here too there was no single major resistance phenotype among the PSM MDR strains. However, there were 11 different resistance profiles among the PSM MDR strains, and the two resistance profiles found for the MRSA strains were TET-CIP-PEN and ERY-TET-CIP-PEN-TYLT.

### 3.2. Multilocus Sequence Typing

The multilocus sequence typing data are presented in Table 3, which provides the sample number, bacteria type and MLST designation with the determined allelic profile.

Six of the seven isolated MRSA strains from the MLT and PSM were determined to be strain ST398 and one strain was ST5.

### 3.3. Disinfectant Susceptibility

The MICs for the 63 *S. aureus* strains, isolated from swine feces determined against 24 disinfectants and disinfectant components, are shown in Table 4. The data for the swine feces set of strains are shown in a table by themselves because these strains differed in their interactions with disinfectants compared to strains isolated from the MLT and PSM, which are shown in Table 5. The *S. aureus* MICs for strains from the MLT and PSM were determined to be higher than the MICs for strains from swine feces against many of the disinfectants, such as DC&R^CP^, CaviCide^CP^, P-128^CP^, P-I, FSS, F25, FS512, OdoBan^CP^, CPB, CPC, CDEAB, CTAB, C8AC, C10AC, C12BAC, C14BAC, and THN. Swine feces strains were 17.5% resistant to chlorhexidine, whereas the group of strains from the MLT and PSM were determined to be 41.6% resistant to chlorhexidine. All strains studied showed no resistance to triclosan. Practically no difference in MICs between the two groups of strains was observed for BKC, C16BAC, formaldehyde, TCC, and Tek-Trol^CP^. The elevated numbers highlighted in yellow in Table 5 show the MICs of six of the seven MRSA strains. The yellow highlighted numbers reflect that 75% of the disinfectants were determined to have increased *S. aureus* MICs in six of the seven MRSA strains. All MRSA strain MICs against the 24 disinfectants and disinfectant components tested are shown in Supplementary Table S4 with the elevated MICs highlighted in yellow. The disinfectant MICs for the 49 *S. aureus* strains isolated from the MLT are shown in Supplementary Table S5. The disinfectant MICs for the 52 *S. aureus* strains isolated from the PSM are shown in Supplementary Table S6.

Table 6 shows the correlation between *S. aureus* strains having increased disinfectant susceptibility with *S. aureus* MDR and MRSA strains. There were no strains from swine feces with elevated susceptibility to disinfectants. However, six (12.3%) of the strains from the MLT and five (9.6%) of the strains from the PSM had increased disinfectant susceptibility levels. There were four MRSA strains among the MLT strains and 3 MRSA strains among the PSM strains. One of the four MRSA strains from the MLT did not have elevated disinfectant susceptibility levels. Therefore, six of the seven MRSA strains had elevated disinfectant susceptibilities. All three MRSA strains isolated from PSM had elevated disinfectant susceptibility levels.

### 3.4. Calculation of Theoretical MICs for Multiple Component Disinfectants

Table 7 lists the calculated ^theo^MICs for the components of DC&R^CP^ and P-128^CP^ against the feces, MLT and PSM strains in comparison to actual MICs for the same components against *S. aureus*. The calculated ^theo^MICs for the BACs, formaldehyde and THN active components of DC&R^CP^ are ^theo^MICs_BACs_^DC&R^, ^theo^MICs_Form_^DC&R^, and ^theo^MICs_THN_^DC&R^ compared to the actual *S. aureus* MICs for swine feces strains against the BACs (C12BAC + C14BAC + C16BAC), formaldehyde, and THN (tris(hydroxylmethyl)nitromethane) found in Table 4. The calculated ^theo^MICs_BACs_^DC&R^, ^theo^MICs_Form_^DC&R^, and ^theo^MICs_THN_^DC&R^ for the active components in DC&R^CP^ compared to the actual *S. aureus* MICs for the MLT and PSM strains against the BACs, formaldehyde, and THN found in Table 5. The calculated ^theo^MICs for the BACs and C10AC active components of P-128^CP^ are ^theo^MICs_BACs_^P-128^ and ^theo^MICs_C10AC_^P-128^ and are compared to the actual *S. aureus* MICs for these components, which are found in Table 4 for the BACs and for C10AC against swine feces strains. The calculated ^theo^MICs_BACs_^P-128^ and ^theo^MICs_C10AC_^P-128^ active components of P-128^CP^ are compared to the actual *S. aureus* MICs, which are found in Table 5 for the BACs and for C10AC against the MLT and PSM strains. The actual MICs_BACs_^DC&R^, MICs_THN_^DC&R^, MICs_BACs_^P-128^, and MICs_C10AC_^P-128^ are slightly higher for the MLT and PSM strains than for the swine feces strains, while the MICs_Form_^DC&R^ are generally unchanged. For the disinfectant DC&R^CP^ the calculated ^theo^MICs_BACs_^DC&R^ from the feces strains or from the MLT and PSM strains are in general relatively similar to the actual MICs necessary for *S. aureus* inhibition by the BACs seen in Table 4 and Table 5. The calculated ^theo^MICs_Form_^DC&R^ and ^theo^MICs_THN_^DC&R^ MICs for the swine feces strains or MLT and PSM strains are not similar to the actual MICs for formaldehyde and THN necessary for inhibition of *S. aureus*. For the disinfectant P-128^CP^, the calculated ^theo^MICs_BACs_^P-128^ from the feces strains, MLT and PSM strains are not high enough to inhibit most of the *S. aureus* strains tested (Table 7). However, the calculated ^theo^MICs_C10AC_^P-128^ are similar to the levels of C10AC necessary for inhibition of *S. aureus*.

### 3.5. Staphylococcus aureus Inhibition by Ammonium Chloride Disinfectant Components

**Figure 1:**
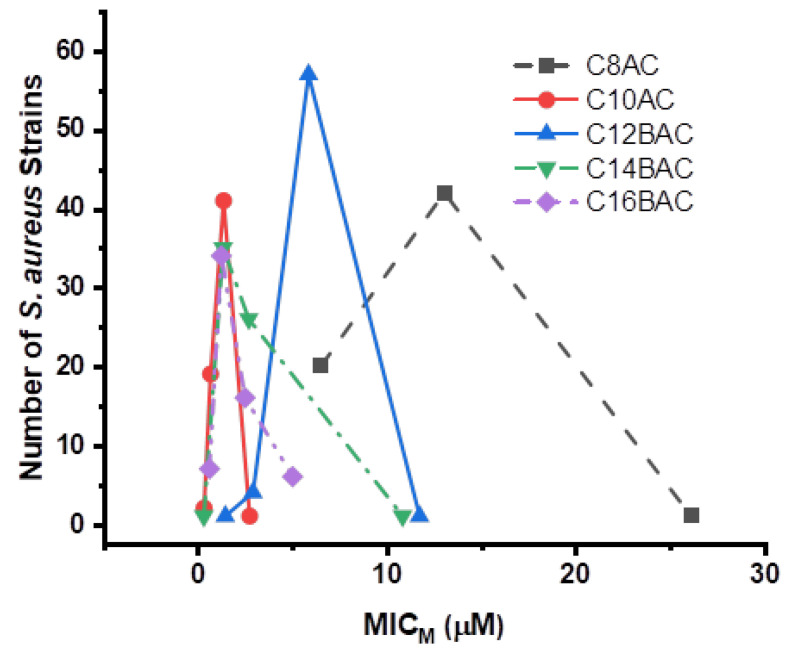
The number of *Staphylococcus aureus* strains at each molar MIC (MIC_M_) for the 63 *Staphylococcus aureus* strains isolated from swine feces against five ammonium chloride disinfectant components.

**Figure 2:**
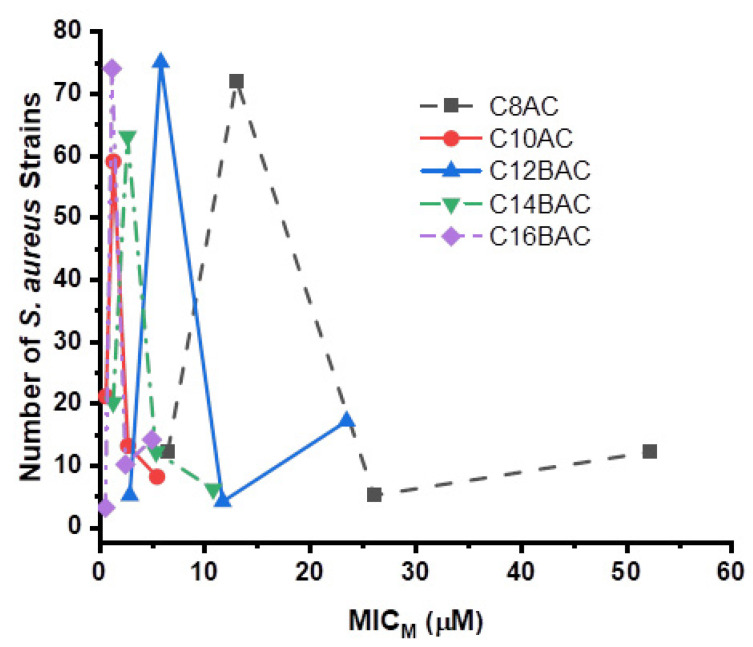
The number of *Staphylococcus aureus* strains at each molar MIC (MIC_M_) for the 101 *Staphylococcus aureus* strains isolated from swine mandibular lymph node tissue and commercial pork sausage meat against five ammonium chloride disinfectant components.

Figure 1 depicts the curves generated for the inhibition of 63 *S. aureus* strains from swine feces by the ammonium chloride disinfectant components C8AC, C10AC, C12BAC, C14BAC, and C16BAC in μmol/L (μM). Figure 2 shows the curves generated for the inhibition of 101 *S. aureus* strains from the MLT and PSM by the ammonium chloride disinfectant components C8AC, C10AC, C12BAC, C14BAC, and C16BAC in μM. C10AC and C16BAC were equally the most effective disinfectant components against *S. aureus*. While C8AC, C10AC, and C12BAC required progressively higher levels to inhibit some of the MLT and PSM strains than for the swine feces *S. aureus* strains.

## 4. Discussion

The 164 *S. aureus* strains isolated from swine feces, MLT, and PSM demonstrated no AMR to daptomycin, nitrofurantoin, linezolid, and tigecycline. These same strains showed very low AMR prevalence to gentamicin, streptomycin, chloramphenicol, vancomycin, and quinupristin/dalfopristin antimicrobials. Some strains showed AMR to ciprofloxacin; however, high AMR was demonstrated among the strains against erythromycin, tylosin tartrate (macrolides), penicillin, and tetracycline. A high level of *S. aureus* macrolide resistance has been observed and was suggested to be due to excessive use of the macrolide antibiotics [114]. As many as 60 resistance genes have been identified in *S. aureus* that can confer resistance to different classes of antimicrobials, such as β-lactam antibiotics, tetracyclines, and the macrolides [115]. The resistance profiles of the *S. aureus* strains studied here show swine feces strains had a limited number of resistance profiles compared to either the MLT or PSM strains. The MDR strains from both the MLT and PSM contained all the MRSA strains detected, and pork sausage has been shown to be an excellent growth medium for *S. aureus* [32,37]. Of the *S. aureus* strains isolated from the MLT, 32.7% were MDR and 25.0% of the MDR strains were MRSA. Of the *S. aureus* strains isolated from PSM, 28.8% were MDR and 20.0% of those were MRSA strains. Even though the number of MDR MLT strains were greater than the number of MDR PSM strains, the number of different resistance profiles were greater for the PSM MDR strains (13 profiles) than for the MLT MDR strains (nine profiles). Seven MRSA strains were isolated from the MLT and PSM, but no MRSA strains were observed among the strains isolated from swine feces. It was previously demonstrated that intracellular contamination of the MLT and PSM by MRSA was 8.2% and 5.8%, respectively [98]; suggesting that the contamination levels of MRSA in swine tissues cannot be eliminated.

Multilocus sequence typing determined six of the seven MRSA strains isolated from the MLT and PSM belonged to strain ST398 while one belonged to strain ST5. A new clone of MRSA with the sequence type ST398 was first described in 2005 [45], and the first study showing a direct association between food animal and human carriage of ST398 was in 2010 [116]. The MRSA strain ST398 was demonstrated to be present in pigs [117,118,119] and in pig farmers [48,120]. MRSA strain ST398 was observed in humans and food animals in Central Europe [121], in humans in Northern Austria [122], Canada [123], the Dominican Republic and New York City [124], and in Midwestern U.S. swine and swine workers [48]. MRSA strain ST398 was observed in fresh pork meat in Germany [125] and in general can be found in final meat products if the pigs were colonized with ST398 [4]. Severe endocarditis, pneumonia, blood steam, and other infections can be caused by CA-MRSA ST398 [59,126,127,128,129,130]. Resistance gene analysis of LA-MRSA CC398 has demonstrated commonly found genes in *S. aureus* and other staphylococci but also novel resistance genes have been described [42]. It was observed at a hospital in South Korea that children were only infected at 6.8% with strain ST5, while adults were infected at a rate of 58% with strain ST5 [131].

Fifty-three of the 164 (32.3%) *S. aureus* strains were resistant to chlorhexidine. Chlorhexidine resistance was observed in 17.5% of swine feces strains and 41.6% of the combined MLT and PSM strains, suggesting both swine MLT and PSM strains had a greater chance of becoming chlorhexidine resistant. In previous experiments, *E. coli* O157:H7 strains from cattle demonstrated only 11% resistance to chlorhexidine [92] and 32% of *C. jejuni* strains were resistant to chlorhexidine [70], but 76% of VRE strains were resistant to chlorhexidine [97]. While strains from non-O157 STECs [94], cattle *Salmonella* strains [95] and swine *C. coli* strains [96] were ~90% resistant to chlorhexidine, and *Ps. aeruginosa* [93] and turkey *Salmonella* strains [132] were 100% resistant to chlorhexidine. All 164 *S. aureus* strains were susceptible to triclosan, which is similar as observed for *Salmonella* [95,132], *E. coli* O157:H7 [92], and the non-O157 STEC strains [94]. Our laboratory regularly refers to triclosan as a pseudo-antibiotic since it is synthetic and not a natural product but functions similarly as an antibiotic [96]. Triclosan is described in the literature as a biocide, but functions like an antimicrobial since it has a specific bacterial cellular target [133]. Triclosan inhibits the final enzyme in the fatty-acid biosynthesis elongation cycle, the highly conserved enzyme enoyl-[acyl-carrier-protein] reductase (NADH) [110]. Triclosan is well known to affect efflux pumps and membrane permeability by causing genetic mutations in at least five genes in *E. coli* resulting in MDR [134], and *Ps. aeruginosa* [93], VRE [97], *C. coli* [96], and *C. jejuni* strains [70] are also highly resistant to triclosan.

All 164 *S. aureus* strains were susceptible to BKC. BKC is a biocide commonly used to preserve human ocular medications, and to clean animal wounds and prevent skin infections. BKC can be used for sanitization in the dairy industry, in fisheries, and on poultry farms [135], and can be used as a Covid-19 hand sanitizer [136]; however, any disinfectant that will disrupt a lipid bilayer will cause virus inactivation. In previous studies it was shown that *C. jejuni* [70], *C. coli* [96] and VRE [97] were susceptible to BKC. Some strains of *E. coli* O157:H7 [92], non-O157 STECs [94] and *Salmonella* from cattle [95], and most *Salmonella* strains from turkeys [132] have shown intermediate resistance to BKC, whereas 97.1% of 175 *Ps. aeruginosa* strains were resistant to BKC [93].

The 164 *S. aureus* swine strains had similar susceptibilities for the disinfectants TCC, P-I, THN, and formaldehyde. The MLT and PSM strains had elevated susceptibilities for DC&R^CP^, Tek-Trol^CP^, CaviCide^CP^, P-128^CP^, BKC, FSS, F25, FS512, OdoBan^CP^, CPB, CPC, CDEAB, CTAB, C8AC, C10AC, C12BAC, C14BAC, and C16BAC compared to the swine feces strains. The MLT and PSM MRSA strains showed elevated susceptibilities to all these disinfectants and disinfectant components, except for Tek-Trol^CP^ when compared to the swine feces strains. The MLT and PSM strains have similar disinfectant susceptibilities as was observed in *C. jejuni* [70], *C. coli* [96], and VRE strains [97]. The disinfectant susceptibilities for previously tested *Salmonella* [95,132], *E. coli* O157:H7 [92], and non-O157 STEC strains [94] were two- to four-fold higher than seen here for the MLT and PSM strains. Whereas the susceptibilities for previously tested *Ps. aeruginosa* strains [93] were 32- to 64-fold higher than obtained here for the MLT and PSM strains. The highest susceptibility values observed were for *S. aureus* against P-I. These levels are similar to those observed previously for *C. jejuni* and are in excess of 49- to 98-fold less than the manufacturer suggested application rate of 100,000 µg/mL of a P-I solution directly applied to wound surfaces [70]. We were unable to observe cross-resistance between the antimicrobials tested and the disinfectants.

The number of MRSA strains correlated well with the strains that had increased disinfectant susceptibility. Out of the 164 *S. aureus* strains tested only 17 strains (10.4%) had elevated disinfectant susceptibility levels, and they were found among the MLT and PSM strains. Six of the seventeen strains with elevated disinfectant susceptibility levels were determined to be MRSA strains and were determined to have increased *S. aureus* susceptibility to 18 of 24 (75%) disinfectants tested. There was one MRSA strain among the MLT strains that did not have elevated disinfectant levels. There were two strains among the MLT strains and five strains among the PSM strains that had elevated disinfectant susceptibility but were not MDR. No strains were found with elevated disinfectant susceptibility levels or tested positive for MRSA among the swine feces strains.

When determining which component of DC&R^CP^ with concentration levels that would inhibit *S. aureus*, the calculated ^theo^MICs_THN_^DC&R^, ^theo^MICs_BACs_^DC&R^, and ^theo^MICs_Form_^DC&R^ were compared with the authentic *S. aureus* MICs against the BACs, THN, and formaldehyde. In general, the calculated ^theo^MICs_BACs_^DC&R^ were at the appropriate levels to inhibit the *S. aureus* strains, and the ^theo^MICs_Form_^DC&R^ and ^theo^MICs_THN_^DC&R^ concentrations were too low to inhibit these bacteria. The ^theo^MICs_BACs_^DC&R^, and specifically the levels of ^theo^MICs_C14BAC_^DC&R^ and ^theo^MICs_C16BAC_^DC&R^ were sufficient to inhibit the *S. aureus* strains. Whereas the levels of ^theo^MICs_C12BAC_^DC&R^ were not sufficient to inhibit 17 of 101 strains (16.8%). In previous studies the level of ^theo^MICs_BACs_^DC&R^ in DC&R^CP^ were sufficient to inhibit all pathogenic bacteria previously studied [92,93,94,95,96,97], except *C. jejuni* [70]. In like manner, when determining which component of P-128^CP^ was the active component against *S. aureus* the calculated ^theo^MICs_BACs_^P-128^ and ^theo^MICs_C10AC_^P-128^ were compared to the authentic *S. aureus* MICs for the BACs and C10AC. The calculated levels for the ^theo^MICs_BACs_^P-128^ were not high enough for inhibition of the *S. aureus* strains but the levels of the calculated ^theo^MICs_C10AC_^P-128^ were similar to the levels required for *S. aureus* inhibition. This result was observed for P-128^CP^ against *E. coli* O157:H7 [92], *Ps. aeruginosa* [93], non-O157 STEC [94], *Salmonella* [95], and VRE [97] strains. However, the BACs component of P-128^CP^ was the most active against *C. coli* [96], and the BACs component and C10AC appeared to act equally and synergistically against *C. jejuni* [70].

The potency of the five-ammonium chloride disinfectant components, C8AC, C10AC, C12BAC, C14BAC, and C16BAC was tested against the *S. aureus* strains and determined that C10AC and C16BAC were equally the most effective against *S. aureus*. C14AC, C12BAC, and C8AC, respectively, required progressively higher concentrations to inhibit the *S. aureus* strains. However, some of the MLT and PSM strains required higher levels of components C8AC and C12BAC for inhibition of *S. aureus* than the swine feces strains did. The length of the carbon chain attached to the ammonium chloride for each disinfectant component is incorporated into the abbreviated names of the components, C8, C10, C12, C14, and C16. In previous potency studies C10AC, C12BAC, and C14BAC were clearly the most effective against *C. jejuni* [70] and *C. coli* [96], while C16BAC was the least effective against these two bacteria. Potency studies of disinfectant components against *E. coli* O157:H7 [92], *Ps. aeruginosa* [93], non-O157 STECs [94], *Salmonella* [95], and VRE [97] determined that C10AC was the most effective ammonium chloride disinfectant component against these bacteria.

## 5. Conclusions

A high prevalence of AMR was demonstrated by the 164 *S. aureus* strains to four antimicrobials, erythromycin (50.6%), tylosin tartrate (42.7%), penicillin (72%), and tetracycline (68.9%), and no AMR was detected to daptomycin, nitrofurantoin, linezolid, and tigecycline. The MLT and PSM strains demonstrated a wide array of resistance profiles. MRSA strains were found only in the MDR strains from the MLT (25.0%) and PSM (20.0%), but not among the swine feces strains. Multilocus sequence typing determined six of the seven MRSA strains isolated from the MLT and PSM were strain ST398 while one strain was ST5. About 17.5% of the swine feces strains and 41.6% of the combined MLT and PSM strains were resistant to chlorhexidine. All 164 strains were susceptible to the pseudo-antibiotic triclosan and to BKC. The MLT and PSM strains had elevated susceptibilities for the disinfectants, DC&R^CP^, Tek-Trol^CP^, CaviCide^CP^, P-128^CP^, BKC, FSS, F25, FS512, OdoBan^CP^, CPB, CPC, CDEAB, CTAB, C8AC, C10AC, C12BAC, C14BAC, and C16BAC compared to the swine feces strains. Six of the seven MRSA strains demonstrated increased MICs to 18 of 24 (75%) disinfectants evaluated compared to non-MRSA strains, and they correlated well with increased disinfectant susceptibility. No strains were found with elevated disinfectant susceptibility levels among the swine feces strains. It was determined that the BAC components of DC&R^CP^ were responsible for the inhibition of *S. aureus* strains. The C10AC component in P-128^CP^ was responsible for *S. aureus* inhibition. The C10AC and C16BAC were equally effective against *S. aureus*. Some of the MLT and PSM *S. aureus* strains required higher levels of the components C8AC and C12BAC for inhibition compared to the swine feces strains. Since the *S. aureus* and MRSA strains were found deep within the MLT, this tissue may be a candidate for specialized treatments or even removal from the human consumption market. The use of formaldehyde and THN in the complex disinfectant DC&R^CP^ is questionable since they are not effective against *S. aureus* at the concentrations present in DC&R^CP^, and the inclusion of formaldehyde and THN may result in additional unnecessary chemicals in the environment. This study establishes susceptibility values for *S. aureus* strains from swine feces, mandibular lymph node tissue, and commercial pork sausage against 24 disinfectants. Since it was demonstrated that *S. aureus* and MRSA strains can be found deep within swine lymph node tissue, it may be beneficial for the consumer if raw swine lymph node tissue was not used in food products and pork sausage.

## Figures and Tables

**Table 1:** Antimicrobial resistance profiles among 164 *Staphylococcus aureus* strains isolated from swine feces, swine mandibular lymph node tissue, and commercial pork sausage meat. MIC = minimum inhibition concentration.

Antimicrobial	MIC_50_ (µg/mL)	MIC_90_ (µg/mL)	MIC Range (µg/mL)			No. (%) Resistant	Breakpoint (µg/mL)
Aminoglycosides							
Gentamicin	≤128	≤128	≤128–1028			1 (0.6)	>500
Kanamycin	≤128	≤128	≤128			CDR *	≥64
Streptomycin	≤512	≤512	≤512–2048			4 (2.4)	≥1000
Amphenicols							
Chloramphenicol	8	16	8–>32			2 (3.8)	≥32
Cyclic Lipopeptides							
Daptomycin	≤0.25	0.5	≤0.25–1			0 (0)	>1
Fluoroquinolones							
Ciprofloxacin	0.5	>4	0.12–>4			21 (12.8)	≥1
Glycopeptides							
Vancomycin	0.5	1	0.5–32			1 (0.6)	≥16
Lincosamides							
Lincomycin	>8	>8	≤1–>8			CDR *	≥32
Macrolides							
Erythromycin	>8	>8	≤0.25–>8			83 (50.6)	≥8
Tylosin Tartrate	2	>32	0.5–>32			70 (42.7)	≥20
Nitrofurans							
Nitrofurantoin	16	16	≤0.25–16			0 (0)	≥128
Oxazolidinones							
Linezolid	2	4	≤0.5–4			0 (0)	≥8
Penicillins							
Penicillin	>16	>16	≤0.25–>16			118 (72)	≥16
Streptogramins							
Quinupristin/Dalfopristin	≤0.5	1	≤0.5–32			6 (3.7)	≥4
Tetracyclines							
Tetracycline	>32	>32	≤1–>32			113 (68.9)	≥16
Tigecycline	0.25	0.5	0.06–0.5			0 (0)	>0.5
	**Number of Strains with Resistance to the Number of Antibiotics**						
No. of Antibiotics	0	1	2	3	4	5	8
No. of Strains (%)	6 (3.6)	49 (29.9)	28 (17.1)	28 (17.1)	39 (23.8)	13 (7.9)	1 (0.6)

* CDR = Cannot Determine Resistance with Sensititre™ plate CMV3AGPF.

**Table 2:** Antimicrobial resistance and resistance profiles among 164 *Staphylococcus aureus* strains isolated from swine feces, swine mandibular lymph node tissue, and commercial pork sausage meat.

Sample Type	No. (%) *S. aureus* Strains Isolated	No. Resistant Strains	Resistance Profiles
Swine Feces	63	18	PEN
		3	TET
		7	TET-PEN
		3	ERY-TET-TYLT
	MDR = 32 (50.8%)	31	ERY-TET-PEN-TYLT
	Strains	1	ERY-TET-CHL-PEN-TYLT
Lymph Node Tissue	49	10	PEN
		5	TET
		2	ERY-TET
		3	ERY-PEN
		1	TET-CIP
		1	TET-PEN
		10	ERY-TET-TYLT
		1	ERY-PEN-TYLT
	Strains: 147L, 150L	2	TET-PEN MRSA *
		3	ERY-TET-PEN
25.0% MDR Strains	Strains: 21L, 38L	2	TET-CIP-PEN MRSA *
were MRSA		1	ERY-TET-CIP-PEN
	MDR = 16 (32.7%)	3	ERY-TET-PEN-TYLT
	Strains	2	ERY-TET-CIP-PEN-STR
		3	ERY-TET-CIP-PEN-TYLT
Pork Sausage Meat	52	6	None
		1	CIP
		5	TET
		7	PEN
		1	ERY-TYLT
		1	TET-CHL
		3	TET-CIP
		7	TET-PEN
		4	ERY-PEN-TYLT
		2	ERY-TET-TYLT
		1	ERY-TET-PEN
20.0% MDR Strains	Strain: D16a	1	TET-CIP-PEN MRSA *
were MRSA		1	TET-CIP-PEN
	MDR = 15 (28.8%)	1	ERY-TET-PEN-TYLT
	Strains	1	ERY-TET-TYLT-GEN
		2	ERY-TET-TYLT-SYN
	Strains: D15, D16	2	ERY-TET-CIP-PEN-TYLT MRSA *
		2	ERY-TET-CIP-PEN-TYLT
		1	ERY-TET-CIP-PEN-STR
		2	ERY-TET-PEN-TYLT-SYN
		1	ERY-TET-CIP-CHL-PEN-STR-TYLT-SYN

* MRSA = methicillin-resistant *S. aureus* (resistant to cefoxitin and oxacillin) [98]; CHL, chloramphenicol; CIP, ciprofloxacin; ERY, erythromycin; GEN, gentamicin; PEN, penicillin; STR, streptomycin; SYN, quinupristin/dalfopristin; TET, tetracycline; TYLT, tylosin tartrate.

**Table 3:** Molecular typing of MRSA (methicillin-resistant *Staphylococcus aureus*) strains isolated from swine mandibular lymph node tissue and commercial pork sausage meat.

Sample	Bacteria	MLST Number (Allelic Profile)
21L	Wildtype *S. aureus*	398 (03-35-19-02-20-26-39)
38L	Wildtype *S. aureus*	398 (03-35-19-02-20-26-39)
147L	Wildtype *S. aureus*	398 (03-35-19-02-20-26-39)
150L	Wildtype *S. aureus*	398 (03-35-19-02-20-26-39)
D15	Wildtype *S. aureus*	5 (01-04-01-04-12-01-10)
D16a	Wildtype *S. aureus*	398 (03-35-19-02-20-26-39)
D16	Wildtype *S. aureus*	398 (03-35-19-02-20-26-39)
Control	*S. aureus* ATCC^®^ 43300	39 (02-02-02-02-02-02-02)
Control	*S. aureus* ATCC^®^ 29213	5 (01-04-01-04-12-01-10)

**Table 4:** Distribution of disinfectant susceptibility profiles for 63 *Staphylococcus aureus* strains isolated from swine feces. MIC = minimum inhibition concentration.

	MIC (µg/mL)																					MIC_50_	MIC_90_
Disinfectant *	0.008	0.0156	0.031	0.0625	0.125	0.25	0.5	1	2	4	8	16	32	64	128	256	512	1024	2048	4096	8192	µg/mL	µg/mL
DC&R^CP^									6 †	29	28											4	8
Tek-Trol^CP^													15	48								64	64
CaviCide^CP^														25	38							128	128
Chlorhexidine ‡							52	10 §	1													0.5	1 §
Triclosan		1	15	26	17	3	1															0.062	0.125
TCC					3	53	7															0.25	0.5
P-128^CP^						3	54	6														0.5	0.5
BKC							12	44	6	1												1	2
P-I																	1	14	22	24	2	2048	4096
FSS						3	45	15														0.5	1
F25						1	43	18	1													0.5	1
FS512						5	44	14														0.5	1
OdoBan^CP^							32	31														0.5	1
CPB					3	29	27	4														0.25	0.5
CPC				1	3	31	24	4														0.25	0.5
CDEAB					1	7	34	21														0.5	1
CTAB						1	30	28	4													1	1
C8AC ¶									20	42	1											4	4
C10AC ¶					2	19	41	1														0.5	0.5
C12BAC ¶							1	4	57	1												2	2
C14BAC ¶					1		35	26		1												0.5	1
C16BAC ¶						7	34	16	6													0.5	1
THN ¶															1	60	2					256	256
Formaldehyde ¶												1	18	43	1							64	64

* Disinfectant and disinfectant component abbreviations: BKC, benzalkonium chloride; chlorhexidine, Novasan Solution^CP^; CPB, cetylpyridinium bromide hydrate; CPC, cetylpyridinium chloride hydrate; CDEAB, ethylhexadecyldimethylammonium bromide; CTAB, cetyltrimethylammonium bromide; FS512, Final Step 512 Sanitizer^CP^; FSS, Food Service Sanitizer^CP^; F25, F-25 Sanitizer^CP^; P-I, providone-iodine^CP^; C8AC, dioctyldimethylammonium chloride; C10AC, didecyldimethylammonium chloride; C12BAC, benzyldimethyldodecylammonium chloride; C14BAC, benzyldimethyltetradecylammonium chloride; C16BAC, benzyldimethylhexadecylammonium chloride; TCC, triclocarban; THN, tris(hydroxylmethyl)nitromethane; and ^CP^ = commercial product. † Number of strains at this MIC. ‡ MICs ≥1 μg/mL are considered resistant to chlorhexidine [111]. § The entries in red indicate resistance. ¶ This entry is a disinfectant component.

**Table 5:** Distribution of disinfectant susceptibility profiles for 101 *Staphylococcus aureus* strains isolated from swine mandibular lymph node tissue and commercial pork sausage meat. MIC = minimum inhibition concentration.

	MIC (µg/mL)																					MIC_50_	MIC_90_
Disinfectant *	0.008	0.0156	0.031	0.0625	0.125	0.25	0.5	1	2	4	8	16	32	64	128	256	512	1024	2048	4096	8192	µg/mL	µg/mL
DC&R^CP^									2 †	26	53	6 ‡	14									8	32
Tek-Trol^CP^													34	53	14							64	128
CaviCide^CP^													2	27	55	12	5					128	256
Chlorhexidine §							59	42 ¶														0.5	1 ¶
Triclosan	1		4	11	44	34	7															0.125	0.25
TCC				1	15	72	13															0.25	0.5
P-128^CP^						1	64	23	13													0.5	2
BKC							3	54	26	16	2											1	4
P-I																		1	44	56		4096	4096
FSS							32	49	16	2	2											1	2
F25							42	41	16	2												1	2
FS512							31	53	16	1												1	2
OdoBan^CP^							19	63	11	6	1		1									1	2
CPB					1	33	49	1	1	16												0.5	4
CPC					2	32	45	5		17												0.5	4
CDEAB				1	1	3	51	28		16	1											0.5	4
CTAB						2	16	64	2	12	5											1	4
C8AC **									12	72	5	12										4	16
C10AC **						21	59	13	8													0.5	1
C12BAC **								5	75	4	17											2	8
C14BAC **							20	63	12	6												1	2
C16BAC **						3	74	10	14													0.5	2
THN **															2	78	20	1				256	512
Formaldehyde **													2	99								64	64

* Disinfectant and disinfectant component abbreviations: BKC, benzalkonium chloride; chlorhexidine, Novasan Solution^CP^; CPB, cetylpyridinium bromide hydrate; CPC, cetylpyridinium chloride hydrate; CDEAB, ethylhexadecyldimethylammonium bromide; CTAB, cetyltrimethylammonium bromide; FS512, Final Step 512 Sanitizer^CP^; FSS, Food Service Sanitizer^CP^; F25, F-25 Sanitizer^CP^; P-I, providone-iodine^CP^; C8AC, dioctyldimethylammonium chloride; C10AC, didecyldimethylammonium chloride; C12BAC, benzyldimethyldodecylammonium chloride; C14BAC, benzyldimethyltetradecylammonium chloride; C16BAC, benzyldimethylhexadecylammonium chloride; TCC, triclocarban; THN, tris(hydroxylmethyl)nitromethane; and ^CP^ = commercial product. † Number of strains at this MIC. ‡ The numbers highlighted in yellow show the MICs of 6 of 7 MRSA strains. § MICs ≥1 µg/mL are considered resistant to chlorhexidine [111]. ¶ The entries in red indicate resistance. ** This entry is a disinfectant component.

**Table 6:** Correlation between MDR and MRSA strains and strains showing elevated disinfectant susceptibility isolated from swine feces, swine mandibular lymph node tissue, and commercial pork sausage meat. MDR = multidrug-resistant, MRSA = methicillin-resistant *Staphylococcus aureus*.

Sample Type	No. of MDR * *S. aureus* Strains	No. of MRSA Strains †	No. of Strains with Elevated Disinfectant Susceptibility ‡
Swine Feces	None	None	None
Lymph Node Tissue	+	+	−
	+	+	+ §, ¶
	+	+	+ §, **
	+	+	+ §, ¶, **
	+	−§§	+ §
	+	−	+ ¶
	2 −‡‡	−	2 + §
Semi-totals	6 (12.3%)	4 strains	7 strains
Pork Sausage Meat	+	+	+ §
	+	+	+ ††
	+	+	+ §, **, ††
	2+	−§§	2 +
	4 −	−	4 +
	−	−	+ §, ¶, **
Semi-totals	5 (9.6%)	3 strains	10 strains
Totals	11 strains	7 strains	17 strains

* MDR strains are resistant to 3 or more classes of antimicrobials. † The positive (+) strains were determined to be MRSA by traditional cefoxitin and oxacillin susceptibility tests and by polymerase chain reaction (PCR) methods [103]. ‡ Susceptibilities for the following 17 disinfectants or disinfectant components were elevated in the positive (+) disinfectant susceptibility strains: BKC, benzalkonium chloride; CaviCide^CP^; P-128^CP^; CPB, cetylpyridinium bromide hydrate; DC&R^CP^; CDEAB, ethylhexadecyldimethylammonium bromide; F25, F-25 Sanitizer^CP^; FS512, Final Step 512 Sanitizer^CP^; FSS, Food Service Sanitizer^CP^; CPC, cetylpyridinium chloride hydrate; CTAB, cetyltrimethylammonium bromide; OdoBan^CP^; and the disinfectant components, C8AC, dioctyldimethylammonium chloride; C10AC, didecyldimethylammonium chloride; C12BAC, benzyldimethyldodecyammonium chloride; C14BAC, benzyldimethyltetradecylammonium chloride; C16BAC, benzyldimethylhexadecylammonium chloride. The susceptibility for: § C10AC was not elevated in this strain; ¶ P-128^CP^ was not elevated in this strain; ** C16BAC was not elevated in this strain; and †† CaviCide^CP^ was not elevated in this strain. ‡‡ (–) = Not an MDR *S. aureus* strain. §§ (–) = Not an MRSA strain.

**Table 7:** Comparison of the calculated theoretical MICs (^theo^MICs) for components of DC&R^CP^ and P-128^CP^ to the actual MIC levels of components required for inhibition of 63 *Staphylococcus aureus* strains isolated from feces to 101 *S. aureus* strains from swine mandibular lymph node tissue and commercial pork sausage meat in μg/mL. MIC = minimum inhibition concentration.

DC&R Component MICs—Feces Strains			DC&R Component MICs—Tissue & Sausage		
Component	Calculated ^theo^MICs	Actual MICs from Table 3 *	Component	Calculated ^theo^MICs	Actual MICs from Table 4 †
^theo^BACs^DC&R^	0.25 (9.6%) ‡	0.125 (0.5%) ‡	^theo^BACs^DC&R^	0.25 (2.0%) ‡	0.25 (1.0%) ‡
	0.5 (46.0%)	0.25 (3.7%)		0.5 (25.7%)	0.5 (31.0%)
	1.0 (44.4%)	0.5 (37.0%)		1.0 (52.5%)	1.0 (25.8%)
		1.0 (24.3%)		2.0 (5.9%)	2.0 (33.3)
		2.0 (33.3%)		4.0 (13.9%)	4.0 (3.3%)
		4.0 (1.1%)			8.0 (5.6%)
^theo^Form^DC&R^	0.19 (9.5%) ‡	16.0 (1.6%) ‡	^theo^Form^DC&R^	0.19 (2.0%) ‡	32.0 (2.0%) ‡
	0.37 (46.0%)	32.0 (28.6%)		0.37 (25.7%)	64.0 (98.0%)
	0.74 (44.4%)	64.0 (68.2%)		0.74 (52.5%)	
		128.0 (1.6%)		1.49 (5.9%)	
				2.97 (13.9%)	
^theo^THN^DC&R^	1.56 (9.5%) ‡	128.0 (1.6%) ‡	^theo^THN^DC&R^	1.56 (2.0%) ‡	128.0 (2.0%) ‡
	3.13 (46.0%)	256.0 (95.2%)		3.13 (25.7%)	256.0 (77.2%)
	6.25 (44.4%)	512.0 (3.2%)		6.25 (52.5%)	512.0 (19.8%)
				12.51 (5.9%)	1024.0 (1.0%)
				25.0 (13.9%)	
**P-128 Component MICs—Feces Strains**			**P-128 Component MICs—Tissue & Sausage Strains**		
^theo^BACs^P-128^	0.1 (4.8%) ‡	0.125 (0.5%) ‡	^theo^BACs^P-128^	0.1 (1.0%) ‡	0.25 (1.0%) ‡
	0.2 (85.7%)	0.25 (3.7%)		0.2 (63.4%)	0.5 (31.0%)
	0.4 (9.5%)	0.5 (37.0%)		0.4 (22.8%)	1.0 (25.7%)
		1.0 (24.3%)		0.8 (12.9%)	2.0 (33.3%)
		2.0 (33.3%)			4.0 (3.3%)
		4.0 (1.1%)			8.0 (5.6%)
^theo^C10AC^P-128^	0.15 (4.8%) ‡	0.125 (3.2%) ‡	^theo^C10AC^P-128^	0.15 (1.0%) ‡	0.25 (20.8%) ‡
	0.3 (85.7%)	0.25 (30.1%)		0.3 (63.4%)	0.5 (58.4%)
	0.6 (9.5%)	0.5 (65.1%)		0.6 (22.8%)	1.0 (12.9%)
		1.0 (1.6%)		1.2 (12.9%)	2.0 (7.9%)

* Component MICs obtained from Table 4 for the BACs, (C12BAC, C14BAC, and C16BAC), Form (formaldehyde), THN (tris(hydroxylmethyl)nitromethane), and C10AC (didecyldimethylammonium chloride) against *S. aureus* strains from feces. † Component MICs obtained from Table 5 for the BACs, (C12BAC, C14BAC, and C16BAC), formaldehyde, THN (tris(hydroxylmethyl)nitromethane), and C10AC (didecyldimethylammonium chloride) against *S. aureus* strains from the MLT and PSM. ‡ Percentage of strains at the indicated MIC.

## Data Availability

All data are presented within the text and in the Supplementary Material.

## Supplementary Materials

The following are available online at https://www.mdpi.com/article/10.3390/microorganisms9112401/s1, Table S1: Antimicrobial resistance profiles among 63 *Staphylococcus aureus* strains isolated from swine feces, Table S2: Antimicrobial resistance profiles among 49 *Staphylococcus aureus* strains isolated from swine mandibular lymph node tissue, Table S3: Antimicrobial resistance profiles among 52 *Staphylococcus aureus* strains isolated from commercial pork sausage meat, Table S4: Distribution of disinfectant and disinfectant components susceptibility profiles for the 7 MRSA strains isolated from swine mandibular lymph node tissue and pork sausage meat, Table S5: Distribution of disinfectant and disinfectant component susceptibility profiles for 49 *Staphylococcus aureus* strains isolated from swine mandibular lymph node tissue, Table S6: Distribution of disinfectant and disinfectant component susceptibility profiles for 52 *Staphylococcus aureus* strains isolated from commercial pork sausage meat.
