# Supplementary material for: Disinfectant and Antimicrobial Susceptibility Studies of Staphylococcus aureus Strains and ST398-MRSA and ST5-MRSA Strains from Swine Mandibular Lymph Node Tissue, Commercial Pork Sausage Meat and Swine Feces
Source: Microorganisms. 2021 Nov 22;9(11):2401. doi: 10.3390/microorganisms9112401 (PMC8621428; doi:10.3390/microorganisms9112401)
Supplement: Supplementary file 1 [file microorganisms-09-02401-s001.zip › Table S1.pdf]

Table S1. Antimicrobial resistance profiles among 63 *Staphylococcus aureus* strains isolated from swine feces. MIC = minimum inhibition concentration.

Antimicrobial	MIC_{50} (μg/mL)	MIC_{90} (μg/mL)	MIC Range (μg/mL)	No. (%) Resistant	Breakpoint
**Aminoglycosides**					
Gentamicin	≤ 128	≤ 128	≤ 128 – 128	0 (0)	>500
Kanamycin	≤ 128	≤ 128	≤ 128	CDR*	≥64
Streptomycin	≤ 512	≤ 512	≤ 512	0 (0)	≥1000
**Amphenicols**					
Chloramphenicol	8	16	4 – >32	1 (1·6)	≥32
**Cyclic Lipopeptides**					
Daptomycin	≤ 0.25	≤ 0.25	≤ 0.25 – 0.5	0 (0)	>1
**Fluoroquinolones**					
Ciprofloxacin	0.25	0.5	0.25 – 0.5	0 (0)	≥1
**Glycopeptides**					
Vancomycin	0.5	1	≤ 0.25 – 1	0 (0)	≥16
**Lincosamides**					
Lincomycin	>8	>8	≤ 1 – >8	CDR*	≥32
**Macrolides**					
Erythromycin	>8	>8	≤ 0.25 – >8	35 (55.6)	≥8
Tylosin Tartrate	>32	>32	1 – >32	35 (55.6)	≥20
**Nitrofurans**					
Nitrofurantoin	16	16	16	0 (0)	≥128
**Oxazolidinones**					
Linesolid	2	2	≤ 0.5 – 4	0 (0)	≥8
**Penicillins**					
Penicillin	>16	>16	≤ 0.25 – >16	57 (90.5)	≥16
**Streptogramins**					
Quinupristin/ Dalfopristin	≤ 0.5	≤ 0.5	≤ 0.5 – 2	1 (1.6)	≥4
**Tetracyclines**					
Tetracycline	>32	>32	≤ 1 – >32	45 (71·4)	≥16
Tigecycline	0.25	0.5	0.06 – 0.5	0 (0)	>0·5
	**Number of *S. aureus* strains with resistance against the number of antibiotics**				
No. of Antibiotics	1	2	3	4	5
No. of Strains (%)	21 (33.3)	7 (11.1)	3 (4.8)	31 (49.2)	1 (1.6)

*CDR = Cannot Determine Resistance with the Sensititre™ plate CMV3AGPF.
