# Supplementary material for: Disinfectant and Antimicrobial Susceptibility Studies of Staphylococcus aureus Strains and ST398-MRSA and ST5-MRSA Strains from Swine Mandibular Lymph Node Tissue, Commercial Pork Sausage Meat and Swine Feces
Source: Microorganisms. 2021 Nov 22;9(11):2401. doi: 10.3390/microorganisms9112401 (PMC8621428; doi:10.3390/microorganisms9112401)
Supplement: Supplementary file 1 [file microorganisms-09-02401-s001.zip › Table S2.pdf]

Table S2. Antimicrobial resistance profiles among 49 *Staphylococcus aureus* strains isolated from swine mandibular lymph node tissue. MIC = minimum inhibition concentration.

Antimicrobial	MIC_{50} (µg/mL)	MIC_{90} (µg/mL)	MIC Range (µg/mL)	No. (%) Resistant	Breakpoint
**Aminoglycosides**					
Gentamicin	≤ 128	≤ 128	≤ 128	0 (0)	>500
Kanamycin	≤ 128	≤ 128	≤ 128	CDR*	≥64
Streptomycin	≤ 512	≤ 512	≤ 512 – 1024	2 (4.1)	≥1000
**Amphenicols**					
Chloramphenicol	8	16	4 – 16	0 (0)	≥32
**Cyclic Lipopeptides**					
Daptomycin	≤ 0.25	≤ 0.25	≤ 0.25 – 0.5	0 (0)	>1
**Fluoroquinolones**					
Ciprofloxacin	0.5	1	0.12 – >4	9 (18.4)	≥1
**Glycopeptides**					
Vancomycin	0.5	1	0.5 – 1	0 (0)	≥16
**Lincosamides**					
Lincomycin	>8	>8	≤ 1 – >8	CDR*	≥32
**Macrolides**					
Erythromycin	>8	>8	≤ 0.25 – >8	28 (57.1)	≥8
Tylosin Tartrate	2	>32	0.5 – >32	17 (34.7)	≥20
**Nitrofurans**					
Nitrofurantoin	16	16	16	0 (0)	≥128
**Oxazolidinones**					
Linesolid	2	2	1 – 4	0 (0)	≥8
**Penicillins**					
Penicillin	>16	>16	≤ 0.25 – >16	31 (63.3)	≥16
**Streptogramins**					
Quinupristin/ Dalfopristin	≤ 0.5	1	≤ 0.5 – 2	0 (0)	≥4
**Tetracyclines**					
Tetracycline	>32	>32	≤ 1 – >32	35 (71.4)	≥16
Tigecycline	0.25	0.25	0.06 – 0.5	0 (0)	>0.5
	**Number of *S. aureus* strains with resistance against the number of antibiotics**				
No. of Antibiotics	1	2	3	4	5
No. of Strains (%)	15 (30.6)	9 (18.4)	16 (32.7)	4 (8.2)	5 (10.2)

*CDR = Cannot Determine Resistance with the Sensititre™ plate CMV3AGPF.
