# Supplementary material for: Disinfectant and Antimicrobial Susceptibility Studies of Staphylococcus aureus Strains and ST398-MRSA and ST5-MRSA Strains from Swine Mandibular Lymph Node Tissue, Commercial Pork Sausage Meat and Swine Feces
Source: Microorganisms. 2021 Nov 22;9(11):2401. doi: 10.3390/microorganisms9112401 (PMC8621428; doi:10.3390/microorganisms9112401)
Supplement: Supplementary file 1 [file microorganisms-09-02401-s001.zip › Table S3.pdf]

Table S3. Antimicrobial resistance profiles among 52 *Staphylococcus aureus* strains isolated from commercial pork sausage meat. MIC = minimum inhibition concentration.

Antimicrobial	MIC_{50} (µg/mL)	MIC_{90} (µg/mL)	MIC Range (µg/mL)	No. (%) Resistant	Breakpoint
**Aminoglycosides**					
Gentamicin	≤ 128	≤ 128	≤ 128 – 1028	1 (1.9)	>500
Kanamycin	≤ 128	≤ 128	≤ 128	CDR*	≥64
Streptomycin	≤ 512	≤ 512	≤ 512 – 2048	2 (3.8)	≥1000
**Amphenicols**					
Chloramphenicol	8	16	8 – >32	2 (3.8)	≥32
**Cyclic Lipopeptides**					
Daptomycin	≤ 0.25	0.5	≤ 0.25 – 1	0 (0)	>1
**Fluoroquinolones**					
Ciprofloxacin	0.5	>4	0.25 – >4	12 (23.1)	≥1
**Glycopeptides**					
Vancomycin	1	1	0.5 – 32	1 (1.9)	≥16
**Lincosamides**					
Lincomycin	>8	>8	≤ 1 – >8	CDR*	≥32
**Macrolides**					
Erythromycin	0.5	>8	≤ 0.25 – >8	20 (38.5)	≥8
Tylosin Tartrate	2	>32	1 – >32	18 (34.6)	≥20
**Nitrofurans**					
Nitrofurantoin	16	16	≤ 0.25 – 16	0 (0)	≥128
**Oxazolidinones**					
Linesolid	2	4	2 – 4	0 (0)	≥8
**Penicillins**					
Penicillin	>16	>16	≤ 0.25 – >16	30 (57·7)	≥16
**Streptogramins**					
Quinupristin/ Dalfopristin	≤ 0.5	2	≤ 0.5 – 32	5 (9.6)	≥4
**Tetracyclines**					
Tetracycline	32	>32	≤ 1 – >32	33 (63.5)	≥16
Tigecycline	0.25	0.25	0.06 – 0.5	0 (0)	>0·5

	**Number of *S. aureus* strains with resistance against the number of antibiotics**						
No. of Antibiotics	0	1	2	3	4	5	8
No. of Strains (%)	6 (11.5)	13 (25)	12 (23.1)	9 (17.3)	4 (7.7)	7 (13.5)	1 (1.9)

*CDR = Cannot Determine Resistance with the Sensititre™ plate CMV3AGPF.
