# Supplementary material for: Disinfectant and Antimicrobial Susceptibility Studies of Staphylococcus aureus Strains and ST398-MRSA and ST5-MRSA Strains from Swine Mandibular Lymph Node Tissue, Commercial Pork Sausage Meat and Swine Feces
Source: Microorganisms. 2021 Nov 22;9(11):2401. doi: 10.3390/microorganisms9112401 (PMC8621428; doi:10.3390/microorganisms9112401)
Supplement: Supplementary file 1 [file microorganisms-09-02401-s001.zip › Table S4.pdf]

Table S4. Distribution of disinfectant and disinfectant component susceptibility profiles for the 7 MRSA strains isolated from swine mandibular lymph node tissue and commercial pork sausage meat.

**MRSA Strains**	**Disinfectant* (μg/mL)**																						
	DCR	T-T	CC	Chl	Tric	TCC	P128	BKC	PI	FS	F25	FS5	OB	CPB	CPC	CDE	CTAB	C8	C10	C12	C14	C16	THN	Fom
**21L**	32†	64	512	0.5	0.12	0.5	2	4	‡	8	4	2	32	4	4	4	4	16	1	8	2	1	256	64
**38L**	8	128	64	0.5	0.25	0.25	0.5	1	‡	.5	0.5	1	1	0.25	0.25	0.5	1	2	0.25	2	0.5	0.5	256	64
**147L**	32	32	512	0.5	0.12	0.25	1	4	§	2	2	2	4	4	4	4	4	16	1	8	2	2	256	64
**150L**	32	64	256	0.5	0.12	0.25	1	4	§	2	2	2	2	4	4	4	4	16	1	8	4	1	256	64
**D15**	32	32	512	0.5	0.06	0.25	2	4	§	2	2	2	4	4	4	8	8	16	1	8	4	2	256	64
**D16a**	16	32	128	0.5	0.12	0.25	2	4	§	2	2	2	2	4	4	4	4	8	2	8	2	2	256	64
**D16**	16	32	128	1	0.12	0.25	2	4	§	2	2	2	2	4	4	4	8	8	1	8	2	1	256	64

***Disinfectant and disinfectant component abbreviations: BKC, benzalkonium chloride; CC, CaviCide^{CP}; Chl, Novasan Solution^{CP}; CPB, cetylpyridinium bromide hydrate; CPC, cetylpyridinium chloride hydrate; CDE, ethylhexadecyldimethylammonium bromide; CTAB, cetyltrimethylammonium bromide; DCR, DC&R^{CP}; FS5, Final Step 512 Sanitizer^{CP}; Fom, formaldehyde; FS, Food Service Sanitizer^{CP}; F25, F-25 Sanitizer^{CP}; PI, providone-iodine^{CP}; C8, dioctyldimethylammonium chloride; C10, didecyldimethylammonium chloride; C12, benzyldimethyldodecylammonium chloride; C14, benzyldimethyltetradecylammonium chloride; C16, benzyldimethylhexadecylammonium chloride; P128, P-128^{CP}; OB, OdoBan^{CP}; TCC, triclocarban; THN, tris(hydroxylmethyl)nitromethane; Tric, triclosan; T-T, Tek-Trol^{CP}; and ^{CP} = commercial product. †The numbers highlighted in yellow show the elevated disinfectant MICs for the 7 MRSA strains. ‡The MIC for providone-iodine^{CP} (PI) is 2048 μg/mL. §The MIC for providone-iodine^{CP} (PI) is 4096 μg/mL.
