# Supplementary material for: Disinfectant and Antimicrobial Susceptibility Studies of Staphylococcus aureus Strains and ST398-MRSA and ST5-MRSA Strains from Swine Mandibular Lymph Node Tissue, Commercial Pork Sausage Meat and Swine Feces
Source: Microorganisms. 2021 Nov 22;9(11):2401. doi: 10.3390/microorganisms9112401 (PMC8621428; doi:10.3390/microorganisms9112401)
Supplement: Supplementary file 1 [file microorganisms-09-02401-s001.zip › Table S5.pdf]

Table S5. Distribution of disinfectant and disinfectant component susceptibility profiles for 49 *Staphylococcus aureus* strains isolated from swine mandibular lymph node tissue. MIC = minimum inhibition concentration.

	**MIC (µg/mL)**																					**MIC_{50}**	**MIC_{90}**
Disinfectant*	.008	.0156	.031	.0625	0.125	0.25	0.5	1	2	4	8	16	32	64	128	256	512	1024	2048	4096	8192	**µg/mL**	**µg/mL**
DC&R[CP]									2†	16	21	2	8‡									8	32
Tek-Trol[CP]													16	19	14							64	128
CaviCide[CP]													2	19	19	7	2					128	256
Chlorhexidine§							36	**13**¶														0.5	**1**¶
Triclosan	1		4	8	22	13	1															0.125	0.25
TCC				1	8	33	7															0.5	0.5
P-128[CP]							38	7	4													0.5	1
BKC							3	29	10	6	1											1	4
P-I																			30	19		2048	4096
FSS							18	23	5	1	2											1	2
F25							23	19	6	1												1	2
FS512							15	27	6	1												1	2
OdoBan[CP]							11	31	3	3			1									1	2
CPB					1	15	26			7												0.5	4
CPC					1	17	22	2		7												0.5	4
CDEAB					1	2	26	13		7												0.5	4
CTAB						1	10	31		5	2											1	4
C8AC**									11	31	2	5										4	8
C10AC**						10	30	8	1													0.5	1
C12BAC**								4	35	3	7											2	8
C14BAC**							14	27	6	2												1	2
C16BAC**						2	40	2	5													0.5	2
THN**																33	16					256	512
Formaldehyde**													1	48								64	64

*Disinfectant and disinfectant component abbreviations: BKC, benzalkonium chloride; chlorhexidine, Novasan Solution[CP]; CPB, cetylpyridinium bromide hydrate; CPC, cetylpyridinium chloride hydrate; CDEAB, ethylhexadecyldimethylammonium bromide; CTAB, cetyltrimethylammonium bromide; FS512, Final Step 512 Sanitizer[CP]; FSS, Food Service Sanitizer[CP]; F25, F-25 Sanitizer[CP]; P-I, providone-iodine[CP]; C8AC, dioctyldimethylammonium chloride; C10AC, didecyldimethylammonium chloride; C12BAC, benzyldimethyldodecylammonium chloride; C14BAC, benzyldimethyltetradecylammonium chloride; C16BAC, benzyldimethylhexadecylammonium chloride; TCC, triclocarban; THN, tris(hydroxylmethyl)nitromethane; and [CP] = commercial product. †Number of strains at this MIC. ‡The numbers highlighted in yellow show the MICs for 6 of 7 MRSA strains. §MICs ≥1 µg/mL are considered resistant to chlorhexidine [111]. ¶The entries in **red** indicates resistance. **This entry is a disinfectant component.
