# Supplementary material for: Disinfectant and Antimicrobial Susceptibility Studies of Staphylococcus aureus Strains and ST398-MRSA and ST5-MRSA Strains from Swine Mandibular Lymph Node Tissue, Commercial Pork Sausage Meat and Swine Feces
Source: Microorganisms. 2021 Nov 22;9(11):2401. doi: 10.3390/microorganisms9112401 (PMC8621428; doi:10.3390/microorganisms9112401)
Supplement: Supplementary file 1 [file microorganisms-09-02401-s001.zip › Table S6.pdf]

Table S6. Distribution of disinfectant and disinfectant component susceptibility profiles for 52 *Staphylococcus aureus* strains isolated from commercial pork sausage meat. MIC = minimum inhibition concentration.

	**MIC (µg/mL)**																					**MIC$_{50}$**	**MIC$_{90}$**
Disinfectant*	.008	.0156	.031	.0625	0.125	0.25	0.5	1	2	4	8	16	32	64	128	256	512	1024	2048	4096	8192	**µg/mL**	**µg/mL**
DC&R[CP]										10†	32	4‡	6									8	32
Tek-Trol[CP]													18	34								64	64
CaviCide[CP]														8	36	5	3					128	256
Chlorhexidine§							23	**29**¶														**1**¶	**1**
Triclosan				3	22	21	6															0.25	0.5
TCC					7	39	6															0.25	0.5
P-128[CP]						1	26	16	9													0.5	2
BKC								25	16	10	1											2	4
P-I																		1	14	37		4096	4096
FSS							14	26	11	1												1	2
F25							19	22	10	1												1	2
FS512							16	26	10													1	2
OdoBan[CP]							8	32	8	3	1											1	2
CPB						18	23	1	1	9												0.5	4
CPC					1	15	23	3		10												0.5	4
CDEAB				1		1	25	15		9	1											0.5	4
CTAB						1	6	33	2	7	3											1	4
C8AC**									1	41	3	7										4	16
C10AC**						11	29	5	7													0.5	2
C12BAC**								1	40	1	10											2	8
C14BAC**							6	36	6	4												1	2
C16BAC**						1	34	8	9													0.5	2
THN**															2	45	4	1				256	256
Formaldehyde**													1	51								64	64

*Disinfectant and disinfectant component abbreviations: BKC, benzalkonium chloride; chlorhexidine, Novasan Solution[CP]; CPB, cetylpyridinium bromide hydrate; CPC, cetylpyridinium chloride hydrate; CDEAB, ethylhexadecyldimethylammonium bromide; CTAB, cetyltrimethylammonium bromide; FS512, Final Step 512 Sanitizer[CP]; FSS, Food Service Sanitizer[CP]; F25, F-25 Sanitizer[CP]; P-I, providone-iodine[CP]; C8AC, dioctyldimethylammonium chloride; C10AC, didecyldimethylammonium chloride; C12BAC, benzyldimethyldodecylammonium chloride; C14BAC, benzyldimethyltetradecylammonium chloride; C16BAC, benzyldimethylhexadecylammonium chloride; TCC, triclocarban; THN, tris(hydroxylmethyl)nitromethane; and [CP] = commercial product. †Number of strains at this MIC. ‡The numbers highlighted in yellow show the MICs for 6 of 7 MRSA strains. §MICs ≥1 µg/mL are considered resistant to chlorhexidine [111]. ¶The entries in **red** indicate resistance. **This entry is a disinfectant component.
